# Cost-effectiveness of Financial Incentives for Patients and Physicians to Manage Low-Density Lipoprotein Cholesterol Levels

**DOI:** 10.1001/jamanetworkopen.2018.2008

**Published:** 2018-09-14

**Authors:** Ankur Pandya, David A. Asch, Kevin G. Volpp, Stephen Sy, Andrea B. Troxel, Jingsan Zhu, Milton C. Weinstein, Meredith B. Rosenthal, Thomas A. Gaziano

**Affiliations:** 1 Department of Health Policy and Management, Harvard T. H. Chan School of Public Health, Boston, Massachusetts; 2 Department of Information, Decisions and Operations, The Wharton School, University of Pennsylvania, Philadelphia; 3 Department of Medicine, Perelman School of Medicine, University of Pennsylvania, Philadelphia; 4 Department of Health Care Management, The Wharton School, University of Pennsylvania, Philadelphia; 5 Center for Health Equity Research and Promotion, Cpl Michael J. Crescenz Veterans Affairs Medical Center, Philadelphia, Pennsylvania; 6 Department of Medical Ethics and Health Policy, Perelman School of Medicine, University of Pennsylvania, Philadelphia; 7 Division of Biostatistics, Department of Population Health, New York University School of Medicine, New York; 8 Division of Cardiovascular Medicine, Brigham and Women’s Hospital, Boston, Massachusetts

## Abstract

**Importance:**

Financial incentives shared between physicians and patients were shown to significantly reduce low-density lipoprotein cholesterol (LDL-C) levels in a randomized clinical trial, but it is not known whether these health benefits are worth the added incentive and utilization costs required to achieve them.

**Objective:**

To evaluate the long-term cost-effectiveness of financial incentives on LDL-C level control.

**Design, Setting, and Participants:**

In this economic evaluation, a previously validated microsimulation computer model was parameterized using individual-level data from the randomized clinical trial on financial incentives, National Health and Nutrition Examination Surveys for model population inputs, and other published sources. The study was conducted from April 15, 2016, to March 29, 2018.

**Interventions:**

The following interventions were used: (1) usual care, (2) trial control strategy (increased cholesterol level monitoring and use of electronic pill bottles), (3) financial incentives for physicians, (4) financial incentives for patients, and (5) incentives shared between physicians and patients.

**Main Outcomes and Measures:**

Discounted costs (2017 US dollars), lifetime cardiovascular disease risk, quality-adjusted life-years (QALYs), and incremental cost-effectiveness ratios (ICERs).

**Results:**

The model population (n = 1 000 000 [30.7% women]) had similar mean (SD) age (61.5 [11.9] years) and LDL-C level (153.9 mg/dL) as the observed trial population (n = 1503 [42.7% women]; age, 62.0 [8.7] years; and LDL-C level, 160.6 mg/dL). Using base-case assumptions (including a 10-year waning period of LDL-C level reductions), the usual-care strategy was dominated (higher costs and lower QALYs) by all other strategies. Strategies for physician- or patient-only incentives were dominated by the shared-incentives strategy, which had an ICER of $60 000/QALY compared with the trial control strategy. In a sensitivity analysis regarding the duration of LDL-C level reductions, the shared-incentives strategy remained cost-effective (ICERs <$100 000/QALY and <$150 000/QALY) for scenarios with LDL-C level reductions lasting, with linear waning, at least 7 and 5 years, respectively. In the 1-way sensitivity analysis for the time horizon of the analysis, the ICER of the shared-incentives strategy exceeded $100 000/QALY at 11 years and $150 000/QALY at 8 years. In probabilistic sensitivity analysis, the shared-incentives intervention was cost-effective in 69% to 77% of iterations using cost-effectiveness thresholds of $100 000 to $150 000/QALY. Cost-effectiveness results were also sensitive to the duration of intervention costs.

**Conclusions and Relevance:**

This study suggests that the financial incentives shared between patients and physicians for LDL-C level control meet conventional standards of cost-effectiveness, but these results appeared to be sensitive to assumptions about the durations of LDL-C level reductions and years of intervention costs included, as well as to the choice of time horizon.

## Key Points

**Question:**

Are reductions in cardiovascular disease risk produced by financial incentives placed on patients, physicians, or both to control low-density lipoprotein cholesterol levels worth the costs needed to achieve these health benefits?

**Findings:**

In this model-based economic evaluation, incentives shared between patients and physicians had an incremental cost-effectiveness ratio of $60 000/quality-adjusted life-year. However, this result was sensitive to assumptions around the durations of low-density lipoprotein cholesterol level reductions and incentive payments.

**Meaning:**

Shared incentives between patients and physicians appear to provide reasonable value (<$100 000/quality-adjusted life-year) for the health gains that they produce, although longer-term demonstrations are needed to show how long these low-density lipoprotein cholesterol level reductions persist.

## Teaser

This model-based economic evaluation examines the cost-effectiveness of financial incentives for patients and physicians in controlling low-density lipoprotein (LDL) cholesterol levels.

## Introduction

High cholesterol level is a leading risk factor for mortality and morbidity.^1^ Statins are effective and cost-effective medications for improving cholesterol levels and cardiovascular disease outcomes, but many patients for whom statins are indicated do not receive prescriptions for them, and the long-term adherence rate for patients who have received prescriptions is below 50%.^2,3,4,5^ Reducing population cardiovascular disease risk is within reach, but improving cholesterol levels will require behavior changes from physicians, patients, or both.

Financial incentives, such as pay-for-performance policies, have shown mixed results in changing physician or patient behaviors and improving health outcomes.^6,7,8^ Asch et al^9^ conducted a 4-arm randomized clinical trial and found that financial incentives shared between physicians and patients were superior at reducing low-density lipoprotein cholesterol (LDL-C) levels compared with no financial incentives, and superior to financial incentives delivered to physicians or patients alone. The trial made innovative use of principles of behavioral economics and electronic pill bottle technology to enhance the effect of financial incentives,^10^ but left unanswered whether the LDL-C level reductions represented good value given the added costs of the financial incentives, electronic pill bottles, and increased use of statins.

Health care payers should consider long-term health and cost outcomes when evaluating the value of quality improvement interventions. Therefore, our objective was to use a previously developed and validated cardiovascular disease microsimulation model to evaluate the cost-effectiveness of financial incentives to physicians, patients, or both on LDL-C level reductions based on the findings from the Asch et al^9^ trial. The control group in the trial also received electronic pill bottles and increased monitoring (4 annual lipid level measurements per trial protocol compared with 1 per year per current lipid guidelines) and experienced sustained LDL-C level reductions in the 15-month follow-up period. Because the control group in the trial did not correspond to usual care, we also included a virtual usual-care group in our cost-effectiveness analysis that did not include these elements.

## Methods

### Overview

We used the previously developed Cardiovascular Disease Policy Model for Risk, Events, Detection, Interventions, Costs, and Trends (CVD PREDICT) Model to simulate long-term discounted health and cost consequences (2017 US dollars) of financial incentives for cholesterol level control based on data from the Asch et al^9^ randomized clinical trial and other published sources (Table 1).^11,12,13^ We included a lifetime horizon in our base-case analysis and performed our analysis from a health care payer perspective; we varied the time horizon in a 1-way sensitivity analysis, recognizing that some payers could care about shorter time frames. Per cost-effectiveness analysis modeling recommendations, the model time horizon should be long enough to capture all relevant health and cost outcomes.^14^ Therefore, although we assumed varying durations of LDL-C level reductions given the 12-month intervention in the Asch et al^9^ trial (with follow-up to 15 months), all relevant long-term health outcomes were captured using longer-term (including lifetime in the base-case analysis) model time horizons. For example, an individual in the model who has an averted fatal cardiovascular disease event in year 2 of the model (because of their lower LDL-C level in year 1 of the model) would have relevant incremental health effects in all model years until the individual died of cardiovascular disease or other causes.

**Table 1.** Model Variables Examined in Sensitivity Analyses

Variable	Base-Case Value	Sensitivity Analysis Range	Probability Distribution for Sensitivity Analyses	Source
LDL-C level reduction at 15 mo, mean (SD), mg/dL				
Control group	25.4 (40.0)	0-103.8	Bootstrapped	Asch et al,^9^ 2015
Physician-incentives group	29.6 (39.1)	0-106.1	Bootstrapped	Asch et al,^9^ 2015
Patient-incentives group	23.3 (37.5)	0-96.8	Bootstrapped	Asch et al,^9^ 2015
Shared-incentives group	33.0 (37.2)	0-106.0	Bootstrapped	Asch et al,^9^ 2015
Waning of LDL-C level reduction (years until no benefit)	None	1.0-30.0	Not applicable	Assumption
Relative risks based on 38.7-mg/dL reduction in LDL-C level				
CHD events	0.77	0.73-0.80	Lognormal	Baigent et al,^2^ 2005
Stroke events	0.83	0.78-0.88	Lognormal	Baigent et al,^2^ 2005
Mean medication adherence at 15 mo, %				
Control group	26.9	±15	β Distribution	Asch et al,^9^ 2015
Physician-incentives group	31.4	±15	β Distribution	Asch et al,^9^ 2015
Patient-incentives group	33.4	±15	β Distribution	Asch et al,^9^ 2015
Shared-incentives group	38.6	±15	β Distribution	Asch et al,^9^ 2015
Mean incentives payments (total per-patient 12-mo payments), $				
Control group	0	Not applicable	Not applicable	Asch et al,^9^ 2015
Physician-incentives group	521	0-1200	γ Distribution	Asch et al,^9^ 2015
Patient-incentives group	187	0-1200	γ Distribution	Asch et al,^9^ 2015
Shared-incentives group	419	0-1200	γ Distribution	Asch et al,^9^ 2015
Costs of administering payments (only for incentives groups)	109	0-200	γ Distribution	Asch et al,^9^ 2015
Costs of statins (annual)	276	40-2000	Not applicable	Thomson *Red Book*,^11^ 2014
Costs of electronic pill bottle (annual)	159	50-250	γ Distribution	Asch et al,^9^ 2015
Costs of cholesterol tests (annual, assuming 4 tests/y)	125	40-200	γ Distribution	RBRVS^12^

Abbreviations: CHD, coronary heart disease; LDL-C, low-density lipoprotein cholesterol; RBRVS, resource-based relative value scale.

SI conversion factor: To convert LDL-C to millimoles per liter, multiply by 0.0259.

The model simulated cardiovascular disease progression and events for individuals drawn from a subset of the representative National Health and Nutrition Examination Surveys (NHANESs) population,^13^ defined using the eligibility criteria from the Asch et al^9^ trial. Each individual was simulated through 5 possible strategies: (1) usual care (ie, a virtual control group), (2) the trial control group (which included use of electronic pill bottles and increased monitoring), (3) patient incentives, (4) physician incentives, and (5) shared incentives to patients and physicians. We evaluated the trade-offs between health gains and increased health care spending among these strategies using cost-effectiveness analysis.

The Asch et al^9^ trial protocol was approved by the institutional review boards of the University of Pennsylvania and Geisinger Health System and the partners human research committee at Harvard Vanguard Medical Associates. The present cost-effectiveness analysis was part of the original trial protocol approved, including informed patient consent, by the University of Pennsylvania Institutional Review Board. Patient records were deidentified before our analysis for this article. This study followed the Consolidated Health Economic Evaluation Reporting Standards (CHEERS) reporting guideline. The study was conducted from April 15, 2016, to March 29, 2018.

**Figure 1.**
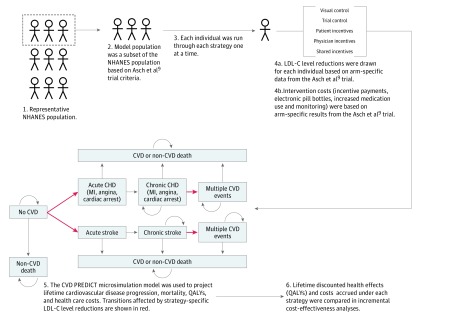
Schematic of Model-Based Cost-effectiveness Analyses The schematic provides an overview of the model population (steps 1 and 2), modeling approach (steps 3-5), and cost-effectiveness analysis (step 6). Individuals started the microsimulation model (step 5) in either the no cardiovascular disease (CVD), chronic coronary heart disease (CHD), or chronic stroke health states depending on their disease history. The curled arrows indicate that simulated patients in the model can stay in the same disease state in the next model cycle. LDL-C indicates low-density lipoprotein cholesterol; MI, myocardial infarction; NHANES, National Health and Nutrition Examination Survey; and QALY, quality-adjusted life-year.

An overview of our methods is shown in Figure 1. eTable 1 in the Supplement describes these CVD PREDICT Model inputs in more detail. Incremental health effects across strategies were driven by strategy-specific levels of LDL-C level reductions, which affected the risk of cardiovascular disease events in the model and were ultimately associated with the differences in the quality-adjusted life expectancies across strategies. Incremental costs across strategies were primarily driven by strategy-specific incentive payments and cost differences from averted cardiovascular disease events.

### Simulation Model and Population

The full description and validation of the CVD PREDICT Model have been published elsewhere.^13^ The CVD PREDICT model is an individual-level state-transition (ie, a microsimulation) model with the following general disease states: disease free, coronary heart disease (divided into myocardial infarction, angina, and cardiac arrest health states), and stroke. Individuals in the model in cardiovascular disease states experience higher mortality risks, lower quality of life, and higher costs relative to those in the disease-free health state. Individuals can experience multiple cardiovascular disease events in the model, which have added acute and chronic health and cost outcomes. The risk of experiencing cardiovascular disease events depends on traditional risk factors (age, sex, smoking status, diabetes history, blood pressure level, cholesterol level, and prior cardiovascular disease history) with further adjustments for treatment status.

The model was populated by weighted sampling (with replacement) of individuals from the 2005-2006, 2007-2008, 2009-2010, and 2011-2012 NHANES surveys subset for the Asch et al^9^ trial eligibility criteria: history of cardiovascular disease (myocardial infarction, angina, or stroke) with LDL-C level greater than 120 mg/dL (to convert to millimoles per liter, multiply by 0.0259) (high-risk participants), history of diabetes with LDL-C level greater than 120 mg/dL (also considered high-risk participants), 10-year coronary heart disease risk greater than 20%, or 10-year coronary heart disease risk greater than 10% with LDL-C level greater than 140 mg/dL (medium-risk participants). All individuals with a history of cancer were excluded.^15^

The CVD PREDICT Model is an individual-level state transition model (ie, a microsimulation model) programmed in Visual C++ 2005 (Microsoft Corp) that uses yearly model cycles. The model updated cardiovascular risk factors (age, total and high-density lipoprotein cholesterol levels, systolic blood pressure, and diabetes status) using previously developed regressions based on NHANES data.^13^ These risk factors were used to estimate the annual risks of cardiovascular disease events (coronary heart disease [cardiac arrest, myocardial infarction, angina] and stroke). These events had acute (ie, 1-year) and postacute (ie, all subsequent years) mortality, morbidity, and health care costs.

### Incentives Strategies

We evaluated the 4 trial arms from the Asch et al^9^ study as well as a virtual control group using conventional cost-effectiveness analysis methods. In the Asch et al study, patients received electronic pill bottles and LDL-C levels were monitored quarterly for 12 months. Patients in the patient- or shared-incentives arm were eligible for daily lottery-based financial payments if they adhered to their statin medications as monitored by the electronic pill bottles ($1022 maximum annual payment). Physicians in the physician-incentives arm were eligible for financial payments ($1024 maximum annual payment) if their patients met LDL-C level targets (individual levels reduced by 10 mg/dL from previous quarter, LDL-C level <100 mg/dL in high-risk patients, or LDL-C level <130 mg/dL in medium-risk patients). The shared-incentives intervention included the physician and patient financial payments set at half the levels in the patient-only and physician-only arms.

The trial control group did not include performance-based financial incentives but did include use of the electronic pill bottles and 4 annual lipid measurements, which is a higher level of monitoring than is standard (1 annual lipid measurement for patients receiving statins). Therefore, we included in our analysis a virtual control group in which individuals did not experience additional changes in cardiovascular risk outside of the usual annual risk factor updates that occur in the background of the CVD PREDICT Model (some of these individuals would also be taking statins, based on national averages and adherence). These risk factor updates were included for all strategies evaluated in the cost-effectiveness analysis.

### Trial-Based LDL-C Level Reductions

We translated trial-based LDL-C level reductions for each incentive strategy to a cardiovascular risk reduction in the CVD PREDICT Model.^2^ The Asch et al^9^ trial deployed incentive payments for 12 months and found sustained LDL-C level reductions at 15 months in all 4 arms 3 months after incentives stopped. We used the observed 15-month LDL-C level reductions to long-term cardiovascular disease risk reductions in our model, which we assumed to wane over time. Specifically, when varying the duration of LDL-C level reduction, we assumed a linear decline of LDL-C level reduction from the end of the first year of the model to a future year (2-30 years) with corresponding adjustments to relative risks for coronary heart disease and stroke. We used a 10-year waning period in our base-case analysis, based on the 10-year decline of treatment outcome for secondary end points (weight, waist circumference, and glycated hemoglobin levels) from the Look AHEAD trial.^16^

We tested the association between this assumption and cost-effectiveness results in sensitivity analyses. LDL-C level reductions 15 months from baseline in the Asch et al^9^ trial ranged from 25.4 mg/dL for the trial control group to 33.0 mg/dL in the shared-incentives group; these absolute LDL-C level reductions were approximately normally distributed among trial participants. In the model-based analyses, we drew individual-level LDL-C level reductions from these normal distributions and converted them to relative risks for coronary heart disease (0.77 per 38.7-mg/dL reduction) and stroke (0.83 per 38.7-mg/dL reduction) based on a large meta-analysis of statin trials.^2^ These relative risks were used to update annual risks of coronary heart disease and stroke in the microsimulation model.

In summary, our model assumes that patients in the virtual control strategy had status quo cardiovascular disease risk, calculated from the risk factors in the NHANES sample used to populate the model cohort, and all other strategies had lowered cardiovascular disease risk based on strategy-specific levels of LDL-C level reductions.

### QALYs and Costs

Health effects for each strategy were quantified using QALYs, which capture both the number and quality of years survived by individuals in the model. Utility values ranging between 0 (death) and 1 (perfect health) were assigned to each health state in the model (disease free, cardiac arrest, myocardial infarction, angina, stroke, and death) and were estimated from the EuroQOL 5 Dimensions questionnaire results from the Medical Expenditure Panel Survey.^17^ We assumed additional disutility from increased statin use associated with taking a pill daily and from incident diabetes for patients who developed statin-induced diabetes.^18,19^ eTable 2 in the Supplement reports the utility values used in the CVD PREDICT Model inputs in more detail. Increased statin use was estimated from intervention-specific adherence results from the Asch et al^9^ trial.

Health care costs were considered from a health care payer perspective. Intervention-specific payments observed in the Asch et al^9^ trial (means of $480, $172, and $386 per patient in the patient, physician, and shared-incentives arms, respectively, which we updated to 2017 US dollars for our analysis) were substantially lower than the maximum possible annual payment ($1024). The cost of administering the incentive payments (including lotteries) was also included for the patient, physician, and shared-incentives strategies ($109); the costs of electronic pill bottles ($159) and lipid testing ($125) were also included for those strategies and the trial control group. We assumed that these costs were included only in the first year of the model in our base-case analysis, but included these costs for longer time periods (2-30 years) in sensitivity analyses.

Acute and postacute costs of cardiovascular disease events and background costs for cardiovascular disease screening and management (including statin drug costs^11^ and cholesterol level testing^12^) were based on estimates from managed care populations in the United States and were included in the cost-effectiveness analyses. eTable 3 in the Supplement reports other cost values used in the CVD PREDICT Model inputs in more detail.

### Base-Case Cost-effectiveness Analysis

We used conventional incremental cost-effectiveness analysis methods to evaluate the 5 strategies included in our study. Lifetime costs and QALYs were discounted at 3% annually per cost-effectiveness analysis recommendations.^20,21^ Incremental cost-effectiveness ratios (ICERs) were calculated for strategies that were not eliminated owing to strong dominance (higher incremental costs and lower incremental QALYs) or weak dominance (lower QALYs but larger ICERs than a more-expensive option). We used cost-effectiveness thresholds of $100 000/QALY and $150 000/QALY to determine the optimal strategy in base-case and sensitivity analyses, which is consistent with the American College of Cardiology–American Heart Association (ACC-AHA) value definition (high value defined as <$50 000/QALY; intermediate value, $50 000/QALY to <$150 000/QALY; and low value, ≥$150 000/QALY).^22,23^

### Sensitivity Analyses

We varied the levels for all variables (or groups of related variables) across plausible ranges or used alternative levels, to assess the robustness of our cost-effective analysis results to changes in these input parameters, paying particular attention to the time horizon of the analysis (1-30 years). We performed two 2-way sensitivity analyses for the shared-incentives strategy: (1) duration of LDL-C level reduction vs years of intervention costs and (2) level of mean incentive costs vs level of LDL-C level reduction (compared with the trial control group). Overall model uncertainty was assessed in a probabilistic sensitivity analysis. In the probabilistic sensitivity analysis, 1000 random values for key model parameters were drawn from prespecified probability distributions (Table 1) for all inputs other than LDL-C level reductions. We used nonparametric bootstrapping of the primary data from the Asch et al^9^ trial to generate 1000 replicates of arm-specific LDL-C level reduction mean (SD) values for the probabilistic sensitivity analysis. The CVD PREDICT Model was programmed in Visual C++ 2005 (Microsoft Corp).

## Results

### Model Population

Characteristics of our model population subset (n = 1 000 000 [30.7% women]) using Asch et al^9^ trial eligibility are reported in eTable 4 in the Supplement. The model population had similar mean (SD) age (61.5 [11.9] years), LDL-C level (153.9 mg/dL), and 10-year coronary heart disease risk (19.2%) as the observed trial population (n = 1503 [42.7% women]; 62.0 [8.7] years; 160.6 mg/dL; and 19.8%, respectively). Compared with the observed trial population, our model population was less likely to include women, African American individuals, or those with a history of coronary heart disease. Model population characteristics were similar using other subset criteria (2013 ACC-AHA cholesterol treatment guidelines or over-weighting individuals with history of coronary heart disease^23^).

### Base-Case Incremental Cost-effectiveness Analysis

The virtual control group had fewer lifetime discounted QALYs and higher lifetime discounted costs compared with all other strategies evaluated in the base-case cost-effectiveness analysis (ie, the virtual control group was strongly dominated). Costs for the virtual control group were higher than other strategies because of lower cost offsets from fewer averted cardiovascular disease events (average lifetime cardiovascular disease incidence of 55.7%) compared with the other strategies (incidence range, 54.5%-54.9%). The patient- and physician-incentive strategies were strongly dominated (more QALYs and lower costs than the shared-incentives strategy) and weakly dominated (higher ICER than the shared-incentives strategy, which was more effective). Compared with the trial control group, the shared-incentives strategy resulted in 0.013 more per-person lifetime discounted QALYs and $807 higher costs, resulting in an ICER of $60 000/QALY. Table 2 reports all of the base-case cost-effectiveness analysis results.

**Table 2.** Lifetime per-Person CVD Events, QALYs, Incentives and Total Costs, and ICERs

Strategy	CVD Events	Life-Years	QALYs^a^	Incentives Costs, $^a^	Total Costs, $^a^	ICER, $
**Base-Case Analysis: 12-mo Intervention Costs; 10-y Waning of LDL-C Level Reduction**						
Trial control group	0.548	19.335	11.648	0	38 909	Reference
Virtual control	0.557	19.251	11.593	0	39 451	Strongly dominated
Patient-incentives group	0.549	19.327	11.641	187	39 554	Strongly dominated
Physician-incentives group	0.547	19.348	11.655	521	39 610	Weakly dominated^b^
Shared-incentives group	0.545	19.359	11.661	419	39 716	60 000/QALY
**Sensitivity Analysis: 12-mo Intervention Costs; 30-y Waning of LDL-C Level Reduction**						
Trial control group	0.534	19.455	11.710	0	38 025	Reference
Shared-incentives group	0.527	19.513	11.741	419	38 591	19 000/QALY
Physician-incentives group	0.530	19.487	11.727	521	38 597	Strongly dominated
Patient-incentives group	0.536	19.437	11.698	187	38 758	Strongly dominated
Virtual control	0.557	19.251	11.593	0	39 451	Strongly dominated
**Sensitivity Analysis: Lifetime Intervention Costs; 10-y Waning of LDL-C Reduction**						
Virtual control	0.557	19.251	11.593	0	39 451	Reference
Trial control group	0.548	19.335	11.648	0	42 827	61 000/QALY
Patient-incentives group	0.549	19.327	11.641	3712	47 404	Strongly dominated
Shared-incentives group	0.545	19.359	11.661	8318	50 612	580 000/QALY
Physician-incentives group	0.547	19.348	11.655	10 345	51 842	Strongly dominated
**Sensitivity Analysis: Lifetime Intervention Costs; 30-y Waning of LDL-C Level Reduction**						
Virtual control	0.557	19.251	11.593	0	39 451	Reference
Trial control group	0.534	19.455	11.710	0	41 960	22 000/QALY
Patient-incentives group	0.536	19.437	11.698	3712	46 643	Strongly dominated
Shared-incentives group	0.527	19.513	11.741	8318	49 553	250 000/QALY
Physician-incentives group	0.530	19.487	11.727	10 345	50 894	Strongly dominated

Abbreviations: CVD, cardiovascular disease; ICER, incremental cost-effectiveness ratio; LDL-C, low-density lipoprotein cholesterol; QALY, quality-adjusted life-year.

^a^ Costs (2017 US dollars) were discounted at 3% annual rate.

^b^ The physician-incentives group had a higher ICER than the shared-incentives group, which is more effective; therefore, the physician-incentives group is weakly dominated and the shared-incentives strategy ICER is compared with the trial control group, per the accepted methods of incremental cost-effectiveness analysis.

### Sensitivity Analyses

Table 2 also provides incremental cost-effectiveness analyses performed using different assumptions for duration of LDL-C level reductions and years of intervention costs included. The base-case analysis assumed 10-year waning of LDL-C level reduction benefits for all trial intervention arms; when LDL-C level benefits waned linearly from the end of the 1-year trial to 30 years, the shared-incentives strategy had an ICER of $19 000/QALY compared with the trial control strategy, which was $5900/QALY in a sensitivity analysis of sustained lifetime LDL-C level reductions without waning. When the duration of LDL-C level reduction benefits lasted 7 or 5 years with linear waning, this ICER was $85 000/QALY and $130 000/QALY, respectively. The base-case analysis also assumed 1-year intervention costs; when intervention costs were included for all years, the shared-incentives strategy had an ICER of $580 000/QALY compared with the trial control strategy, and the virtual control strategy was no longer strongly dominated, resulting in an ICER of $61 000/QALY for the trial control strategy compared with the virtual control strategy.

**Figure 2.**
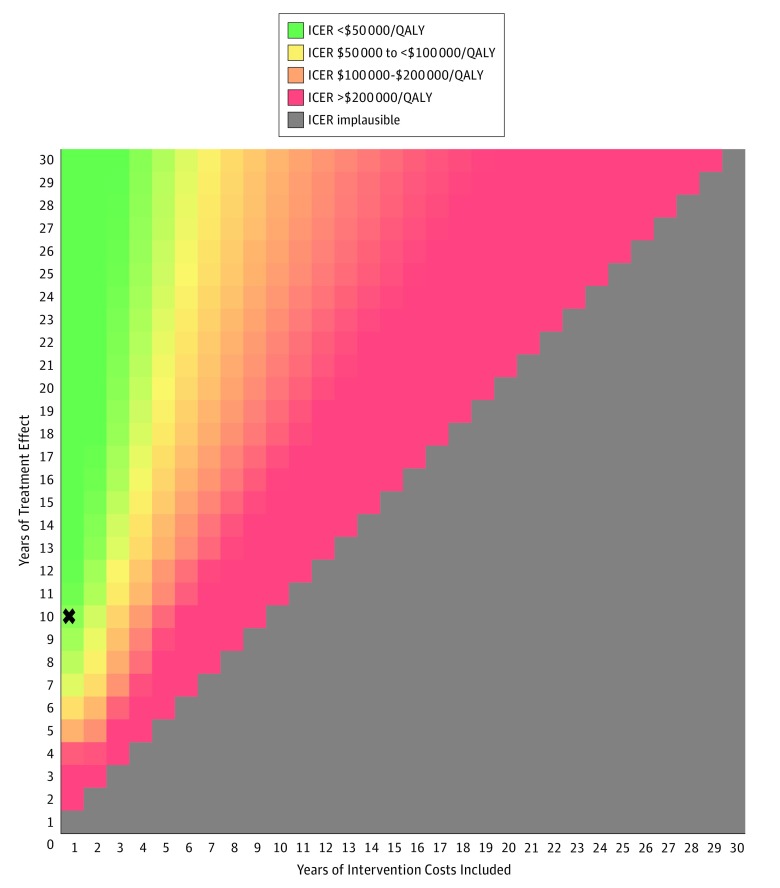
Two-Way Sensitivity Analysis Showing the Incremental Cost-effectiveness Ratios (ICERs) for the Shared-Incentives Strategy Compared With the Trial Control for Different Combinations of Low-Density Lipoprotein Cholesterol Level Reduction Waning and Years of Intervention Costs The regions show combinations of values by color that resulted in ICERs (2017 US dollars) for the shared-incentives strategy compared with the trial control strategy per quality-adjusted life-year (QALY). Implausible results indicate years when intervention costs are included but treatment effects are not observed. The *X* indicates the base-case assumption and result (treatment effect linearly wanes to 0 by year 10).

Figure 2 shows the outcome of varying the duration of LDL-C level reduction benefits (1-30 years) and years of intervention costs included (1-30 years) on the ICER for the shared-incentives strategy compared with the trial control group strategy using a willingness-to-pay (ie, cost-effectiveness threshold) of $100 000/QALY; eFigure 1 in the Supplement shows the same results using a willingness-to-pay of $150 000/QALY. The 1-way sensitivity analysis for the time horizon of the analysis (eFigure 2 in the Supplement) shows the ICER of the shared-incentives strategy exceeding $100 000/QALY at 11 years and $150 000/QALY at 8 years.

**Figure 3.**
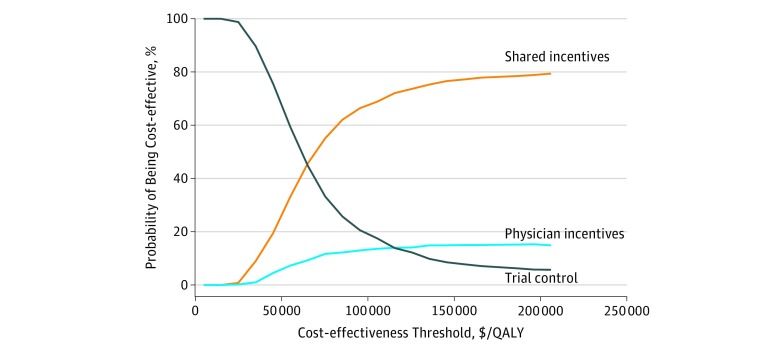
Cost-effectiveness Acceptability Curve for the Probabilistic Sensitivity Analysis The shared-incentives strategy was most likely to be optimal using a willingness-to-pay threshold (2017 US dollars) of $100 000/quality-adjusted life-year (QALY) (69% of probabilistic sensitivity analysis iterations) followed by trial control strategy (18% of probabilistic sensitivity analysis iterations).

eFigure 3 in the Supplement presents a 2-way sensitivity analysis for the same ICER as a joint function of the mean levels of LDL-C level reductions and incentives payments experienced in the shared-incentives strategy. When the benefit of LDL-C level reduction meets or exceeds 29 to 30 mg/dL, the shared-incentives strategy had a favorable ICER (<$100 000/QALY) compared with the trial control strategy. In probabilistic sensitivity analysis (Figure 3), the shared-incentives group was optimal in 69% and 77% of iterations using cost-effectiveness thresholds of $100 000/QALY and $150 000/QALY, respectively. The patient and physician incentives strategies were optimal in 0% and 14% of iterations, respectively, using a cost-effectiveness threshold of $100 000/QALY. The virtual control strategy was not optimal in any iteration. eFigures 4 through 7 in the Supplement show probabilistic sensitivity analysis results for different LDL-C level reduction waning scenarios (5- and 30-year LDL-C level reduction waning, in addition to lifetime LDL-C level reductions) and the combination of 10-year LDL-C level reduction waning with 5 years of intervention costs. When the duration of LDL-C level benefits was 5 years (eFigure 4 in the Supplement), the shared-incentives and trial control strategies were optimal in 27% and 65% of iterations, respectively, using a cost-effectiveness threshold of $100 000/QALY and 52% and 35% of iterations, respectively, using a cost-effectiveness threshold of $150 000/QALY.

## Discussion

In our model-based incremental cost-effectiveness analysis, we found that financial incentives shared between physicians and patients for LDL-C level control produced intermediate value ($60 000/QALY) as defined by the ACC-AHA value framework (ICER $50 000-150 000/QALY) compared with strategies that included financial incentives to either patients or physicians alone and compared with clinical strategies that did not include financial incentives.^22,23^ These base-case results were cost-effective given current US benchmarks for cost-effectiveness (threshold of $100 000/QALY or $150 000/QALY), but these results were sensitive to the duration of the LDL-C level reduction beyond the intervention.

Our base-case analysis assumed a 10-year LDL-C level reduction with linear waning based largely on the Look AHEAD trial, which was the only related trial (for an incentives or lifestyle intervention) that had long-term follow-up (11.5 years) for a shorter-term intervention (6 months to 4 years).^16^ A 2012 review by Jeffery^24^ found that sustained weight loss after financial incentive interventions has been poor, although these studies have typically not been as long (12-month intervention) with their follow-up (3 months) as the Asch et al^9^ trial. The long-run duration of LDL-C level reductions could affect the cost-effectiveness of the shared-incentives strategy. If LDL-C level reductions persisted, with linear waning, for 7 years, the ICER remained below $100 000/QALY and if they persisted for 5 years, the ICER remained below $150 000/QALY. The Asch et al trial followed up patients an additional 3 months after the 12-month intervention was stopped and found no change in the LDL-C level reductions. If these reductions do not wane over time, the shared-incentives strategy had a low ICER ($5900/QALY).

Previous model-based studies have found that statin regimen initiation in eligible individuals (with 10-year cardiovascular disease risk >7.5%, which is a lower-risk group than the population evaluated in this study) met conventional cost-effectiveness standards using generic statin prices.^3,25^ Poor medication adherence remains a challenge, however, driven by patient-, physician-, and system-related factors.^4^ Our study shows that shared financial incentives could be a cost-effective intervention to improve cholesterol level control among high-risk patients. The use of more advanced technologies to guide statin initiation (eg, coronary artery calcium level scoring or genetic test information) or treat high cholesterol levels (eg, proprotein convertase subtilisin/kexin type 9 inhibitors) have been found to exceed conventional cost-effectiveness thresholds (ICERs >$150 000/QALY).^26,27,28^ Because statins are effective, inexpensive at generic prices, and relatively safe, the most cost-effective interventions in cholesterol management will be strategies that improve medication adherence.^29^

### Limitations and Strengths

Our study has several limitations. First, the model extrapolates the observed findings from the Asch et al^9^ trial into future years, in addition to background changes in cardiovascular disease risk factors. These assumptions are needed to generate lifetime estimates of QALYs and costs (the recommended time horizon for cost-effectiveness analyses) and are common for model-based cost-effectiveness analyses based on typical clinical trial follow-up (eg, 12 months).^14,23,25^ We therefore focused our sensitivity analyses on assumptions, such as the duration of LDL-C level reductions, on which projections of trial results are dependent. Second, the findings from the Asch et al trial were from a selected population and might have been more optimistic than results that could be expected in practice owing to observer effects and other deviations from routine care. Those effects, however, likely also apply to the control group in the Asch et al trial and so the net effect is likely to be small and the direction of such bias is hard to estimate.

Third, we performed our analysis from a health care payer perspective. From a societal perspective, incentives payments would be viewed as transfer costs (money changing hands without resource use), which would result in more favorable ICERs for the incentive strategies. Because payers will ultimately decide whether to implement pay-for-performance programs, we used a payer perspective in our study. Fourth, the per-person QALY differences were small, but so were the corresponding cost differences, thus requiring a cost-effectiveness analysis to weigh these trade-offs. On a population level, these differences would translate to important health and cost outcomes given the national prevalence of the targeted population. Fifth, financial incentive policies can lead to gaming and other distortive behaviors. We modeled these effects to the extent that they were captured in the Asch et al^9^ trial, but we did not model any additional adjustments given the lack of data in the published literature to quantify these effects.

This study also has strengths. Our model incorporated original data from a large prospective trial, it was based on credible assumptions about the effects drawn from a larger amount of literature and a comprehensive meta-analysis, it was executed through a validated cardiovascular disease simulation model, and its findings are largely sustained through extensive sensitivity analyses. Given both the promise of the intervention and the importance of duration of LDL-C level reductions on our model-based results, we believe a large-scale demonstration for the shared-incentives strategy with at least 5 years of follow-up is warranted by the Centers for Medicare & Medicaid Services or private health care payers to test this strategy in a real-world setting.

## Conclusions

Our cost-effectiveness analysis answered an important residual question from the Asch et al^9^ trial: were the LDL-C level reductions produced by financial incentives shared between physicians and patients worth the added incentive and utilization costs? Our model-based analyses showed that financial incentives shared between patients and physicians for LDL-C level control met conventional standards of cost-effectiveness. However, these results were sensitive to assumptions about the durations of LDL-C level reductions and years of intervention costs included, prompting the need for a larger, longer-term, real-world demonstration to assess the likelihood that a shared-incentives strategy could be adopted and shown to be effective on a widespread basis.

## Notes

Supplement. **eTable 1.** Disease Progression Inputs Used in the CVD PREDICT Model **eTable 2.** Utilities Used in the CVD PREDICT Model **eTable 3.** Costs (2017 US Dollars) Used in the CVD PREDICT Model **eTable 4**. Trial and Model Baseline Characteristics for Each Strategy (Treatment Arm) **eFigure 1.** Two-Way Sensitivity Analysis Showing the ICER for the Shared Incentives Strategy Compared to the Trial Control for Different Combinations of LDL Reduction Waning and Years of Intervention Costs, Assuming a Cost-effectiveness Threshold of $150,000/QALY **eFigure 2.** One-Way Sensitivity Analysis Showing the ICER for the Shared Incentives Strategy as a Function of Analytical Time Horizon **eFigure 3.** Two-Way Sensitivity Analysis Showing the ICER for the Shared Incentives Strategy Compared to the Trial Control for Different Combinations of LDL Cholesterol Reductions and Average Shared Financial Incentives Payouts **eFigure 4.** Cost-effectiveness Acceptability Curve (CEAC) for the Probabilistic Sensitivity Analysis (PSA) for Scenario of 5-Year LDL Reduction Waning **eFigure 5.** Cost-effectiveness Acceptability Curve (CEAC) for the Probabilistic Sensitivity Analysis (PSA) for Scenario of 10-Year LDL Reduction Waning and Including 5 Years of Intervention Costs **eFigure 6.** Cost-effectiveness Acceptability Curve (CEAC) for the Probabilistic Sensitivity Analysis (PSA) for Scenario of 30-Year LDL Reduction Waning **eFigure 7.** Cost-effectiveness Acceptability Curve (CEAC) for the Probabilistic Sensitivity Analysis (PSA) for Scenario of Lifetime LDL Benefit Duration **eReferences**
