# Supplementary material for: Cost-effectiveness of Financial Incentives for Patients and Physicians to Manage Low-Density Lipoprotein Cholesterol Levels
Source: JAMA Netw Open. 2018 Sep 14;1(5):e182008. doi: 10.1001/jamanetworkopen.2018.2008 (PMC6324619; doi:10.1001/jamanetworkopen.2018.2008)
Supplement: Supplement. — eTable 1. Disease Progression Inputs Used in the CVD PREDICT Model eTable 2. Utilities Used in the CVD PREDICT Model eTable 3. Costs (2017 US Dollars) Used in the CVD PREDICT Model eTable 4. Trial and Model Baseline Characteristics for Each Strategy (Treatment Arm) eFigure 1. Two-Way Sensitivity Analysis Showing the ICER for the Shared Incentives Strategy Compared to the Trial Control for Different Combinations of LDL Reduction Waning and Years of Intervention Costs, Assuming a Cost-effectiveness Threshold of $150,000/QALY eFigure 2. One-Way Sensitivity Analysis Showing the ICER for the Shared Incentives Strategy as a Function of Analytical Time Horizon eFigure 3. Two-Way Sensitivity Analysis Showing the ICER for the Shared Incentives Strategy Compared to the Trial Control for Different Combinations of LDL Cholesterol Reductions and Average Shared Financial Incentives Payouts eFigure 4. Cost-effectiveness Acceptability Curve (CEAC) for the Probabilistic Sensitivity Analysis (PSA) for Scenario of 5-Year LDL Reduction Waning eFigure 5. Cost-effectiveness Acceptability Curve (CEAC) for the Probabilistic Sensitivity Analysis (PSA) for Scenario of 10-Year LDL Reduction Waning and Including 5 Years of Intervention Costs eFigure 6. Cost-effectiveness Acceptability Curve (CEAC) for the Probabilistic Sensitivity Analysis (PSA) for Scenario of 30-Year LDL Reduction Waning eFigure 7. Cost-effectiveness Acceptability Curve (CEAC) for the Probabilistic Sensitivity Analysis (PSA) for Scenario of Lifetime LDL Benefit Duration eReferences [file jamanetwopen-1-e182008-s001.pdf]

Supplementary Online Content

Pandya A, Asch DA, Volpp KG, et al. Cost-effectiveness of financial incentives for patients and physicians to manage low-density lipoprotein cholesterol levels. *JAMA Netw Open*. 2018;1(5):e182008. doi:10.1001/jamanetworkopen.2018.2008

eTable 1. Disease Progression Inputs Used in the CVD PREDICT Model

eTable 2. Utilities Used in the CVD PREDICT Model

eTable 3. Costs (2017 US dollars) Used in the CVD PREDICT Model

eTable 4. Trial and Model Baseline Characteristics for Each Strategy (Treatment Arm)

eFigure 1. Two-Way Sensitivity Analysis Showing the ICER for the Shared Incentives Strategy Compared to the Trial Control for Different Combinations of LDL Reduction Waning and Years of Intervention Costs, Assuming a Cost-effectiveness Threshold of $150,000/QALY

eFigure 2. One-Way Sensitivity Analysis Showing the ICER for the Shared Incentives Strategy as a Function of Analytical Time Horizon

eFigure 3. Two-Way Sensitivity Analysis Showing the ICER for the Shared Incentives Strategy Compared to the Trial Control for Different Combinations of LDL Cholesterol Reductions and Average Shared Financial Incentives Payouts

eFigure 4. Cost-effectiveness Acceptability Curve (CEAC) for the Probabilistic Sensitivity Analysis (PSA) for Scenario of 5-Year LDL Reduction Waning

eFigure 5. Cost-effectiveness Acceptability Curve (CEAC) for the Probabilistic Sensitivity Analysis (PSA) for Scenario of 10-Year LDL Reduction Waning and Including 5 Years of Intervention Costs

eFigure 6. Cost-effectiveness Acceptability Curve (CEAC) for the Probabilistic Sensitivity Analysis (PSA) for Scenario of 30-Year LDL Reduction Waning

eFigure 7. Cost-effectiveness Acceptability Curve (CEAC) for the Probabilistic Sensitivity Analysis (PSA) for Scenario of Lifetime LDL Benefit Duration

eReferences

This supplementary material has been provided by the authors to give readers additional information about their work.

eTable 1. Disease Progression Inputs Used in the CVD PREDICT Model

Parameter	Value	Source
From Disease Free State		
Non-CVD death	Age and sex-specific table	NCHS 2010 [1]
Stroke event	Calibrated risk score	Wolf 1991 [2]
CHD event	Calibrated risk score	Anderson 1991 [3]
% CHD Cardiac Arrest	Age and sex-specific table	Weinstein 1987 [4]
% CHD MI (male)	0.35	NHLBI 2006 [5]
% CHD MI (female)	0.2	NHLBI 2006 [5]
From Stroke state		
Acute (1-year) risk of death	0.15	Carandang 2006 [6]
Chronic (post 1st-year) MI<br>Chronic (post 1st-year) stroke	0.022<br>Calibrated risk score	Touze 2005 [7]<br>Wolf 1991[2]
From MI state		
Acute (1-year) risk of death	0.15	Mozaffarian 2016 [8]
Acute CABG	0.082	Fang 2010 [9]
Acute PTCA	0.3	Fang 2010 [9]
% Procedure death	0.0015	Williams 2006 [10]
Acute 2nd MI (no PTCA)	0.06	Capewell 2006 [11]
Acute 2nd MI (after PTCA)	0.053	Windecker 2014 [12]
Chronic (post 1st-year) repeat MI	0.064	Jokhadar 2004 [13]
Chronic (post 1st-year) repeat MI (with PTCA)	0.052	Jokhadar 2004 [13], Windecker 2014 [12]

From MI and CABG State		
Acute post-CABG death	0.027	Peterson 2004 [14]
Acute 2nd MI	0.047	Windecker 2014 [12]
Repeat MI	0.049	Yusuf 1994 [15], Windecker 2014 [12]
From Angina State		
Acute (1-year) risk of death	0.045	Capewell 2006 [11]
Acute (1-year) risk of cardiac arrest	0.006	Hsia 2008 [16]
Acute (1-year) risk of MI	0.035	Hemingway 2003 [17]
Acute (1-year) risk of MI (with PTCA)	0.031	Hemingway 2003 [17], Windecker 2014 [12]
Acute CABG	0.2	Ford 2007 [18]
Acute PTCA	0.3	Ford 2007 [18]
% Procedure Death	0.0015	Assumption: same as MI
Chronic (post 1st-year) MI	0.035	Assumption: same as acute MI
Chronic (post 1st-year) MI (with PTCA)	0.029	Windecker 2014 [12]
From Angina and CABG state		
Acute post-CABG death	0.027	Assumption: same as MI-CABG
Acute 2nd MI	0.028	Windecker 2014 [12]
Chronic (post 1st-year) MI	0.0278	Hemingway 2003 [17]; Windecker 2014 [12]
From Cardiac Arrest state		
Acute (within 1 year) death	0.954	Nichol 2008 [19]
MI event	0.064	Assumption: same as MI
Chronic (post 1st-year) CVD Mortality		
Proportion of chronic CVD deaths due to CVD	0.28	NHANES-based calculation
Post-stroke all-cause mortality multiplier	2.3	Rosen 2010 [20]
Post-CHD all-cause mortality multiplier (male)	1.6	Smolina 2012 [21]
Post-CHD all-cause mortality multiplier	2.1	Smolina 2012 [21]

(female)		
Post-CHD all-cause mortality >1 event (male)	3.4	Smolina 2012 [21]
Post-CHD all-cause mortality >1 event (female)	2.5	Smolina 2012 [21]

Note: see Pandya et al. 2017 for the full explanation of how these inputs were derived:

Pandya A, Sy S, Cho S, Alam S, Weinstein MC, Gaziano TA. Validation of a Cardiovascular Disease Policy Micro-simulation Model Using Both Survival and Receiver Operating Characteristic Curves. *Med Decis Making*. 2017 Oct;37(7):802-814.

eTable 2. Utilities Used in the CVD PREDICT Model

Parameter	Base-case Value	Base-Case Source
Disease Free	0.877	Sullivan 2006 (15), Mozafarrian 2016 (3)
Chronic Cardiac Arrest	0.808	Sullivan 2006 [22],Taylor 2009 [23]
Chronic MI	0.778	Sullivan 2006 [22]
Chronic MI with CABG	0.778	Sullivan 2006 [22]
Chronic Angina	0.768	Sullivan 2006 [22]
Chronic Angina with CABG	0.768	Sullivan 2006 [22]
Chronic Stroke	0.768	Sullivan 2006 [22]
Utilities for Acute Disease States (disabilities for acute state in parentheses)		
Acute Cardiac Arrest	0.770 (-0.0409)	Sullivan 2006 [22]
Acute MI	0.737 (-0.0409)	Sullivan 2006 [22]
Acute MI with CABG	0.737 (-0.0409)	Sullivan 2006 [22]
Acute Angina	0.727 (-0.0412)	Sullivan 2006 [22]
Acute Angina with CABG	0.727 (-0.0412)	Sullivan 2006 [22]
Acute Stroke	0.716 (-0.0524)	Sullivan 2006 [22]
Disutilities for Events		
Repeat MI	-0.041	Sullivan 2006 [22]
Repeat Stroke	-0.052	Sullivan 2006 [22]
CABG	0	assumption
PTCA	0	assumption
Statin	-0.002	Gage 1996 [24], Hutchins 2015 [25]
Minor statin adverse event	-0.005	Lee 2010 [26] (2 days of life lost)

Major statin adverse event	-0.038	Lee 2010 [26] (2 weeks of life lost)

Note: see Pandya et al. 2017 for the full explanation of how these inputs were derived:

Pandya A, Sy S, Cho S, Alam S, Weinstein MC, Gaziano TA. Validation of a Cardiovascular Disease Policy Micro-simulation Model Using Both Survival and Receiver Operating Characteristic Curves. *Med Decis Making*. 2017 Oct;37(7):802-814.

eTable 3. Costs (2017 US Dollars) Used in the CVD PREDICT Model

Parameter	Base-Case Value	Base-Case Source
Costs for Chronic Disease States		
Disease Free	$0	Assumption: None
Chronic CHD	$3,368	Lee 2010 [26]
Chronic Stroke	$2,225	Pignone 2006 [27]
Costs for Acute Disease States		
Acute Cardiac Arrest	$20,277	O'Sullivan 2011 [28]
Acute MI	$59,301	O'Sullivan 2011 [28]
Acute Angina	$30,660	O'Sullivan 2011 [28]
Acute Stroke	$20,127	O'Sullivan 2011 [28]
Costs for Procedures and Repeat Events		
Repeat MI	$59,301	O'Sullivan 2011 [28]
Repeat Stroke	$20,127	O'Sullivan 2011 [28]
CABG	$38,797	O'Sullivan 2011 [28]
PTCA	$36,556	O'Sullivan 2011 [28]
Screening Costs		
Non-lab test (GP visit in Stage 1)	$79	Pletcher 2009 [29]
Cholesterol (lab) test	$37	Pletcher 2009 [29]
# extra GP visits during Stage 2	1	Assumption
# lab tests/year after treatment	1	Lazar 2011 [30], Expert Opinion
# GP visits/year after treatment	1	Lazar 2011 [30], Expert Opinion
Statin Drug and Adverse Event Costs		
Statin	$281	Redbook 2009 [31]
Anti-hypertensive	$217	Nuckols 2011 [32]
Aspirin	$8	Pignone 2006 [27]
ACE Inhibitor	$55	Shah 2011 [33], Redbook 2009 [31]

Beta Blocker	$55	Shah 2011 [33], Redbook 2009 [31]
Mild adverse event	$188	Lee 2010 [26]
Major adverse event	$7,400	Lee 2010 [26]

Note: see Pandya et al. 2017 for the full explanation of how these inputs were derived:

Pandya A, Sy S, Cho S, Alam S, Weinstein MC, Gaziano TA. Validation of a Cardiovascular Disease Policy Micro-simulation Model Using Both Survival and Receiver Operating Characteristic Curves. *Med Decis Making*. 2017 Oct;37(7):802-814.

eTable 4. Trial and Model Baseline Characteristics for Each Strategy (Treatment Arm)

Characteristic	Trial	Model
n	1,503	1,000,000
Age, mean (SD), years	61.99 (8.7)	61.5 (11.9)
Female sex (%)	42.7	30.7
African American (%)	15.5	10.6
Currently smoking (%)	*	31.8
History of diabetes	18.1	35.2
Systolic blood pressure mean (SD), mmHg	129.1 (14.9)	136.6 (20.8)
Total cholesterol mean (SD), mg/dL	*	228.5 (46.4)
LDL cholesterol mean (SD), mg/dL	160.6 (27.2)	153.9 (39.3)
HDL cholesterol mean (SD), mg/dL	*	44.3 (12.9)
History of coronary heart disease (%)	34.5	7.7
Taking cholesterol medication (%)	33.2	27.3
CHD risk mean (SD)	19.8 (8.7)	19.2 (8.4)

*not included in trial dataset

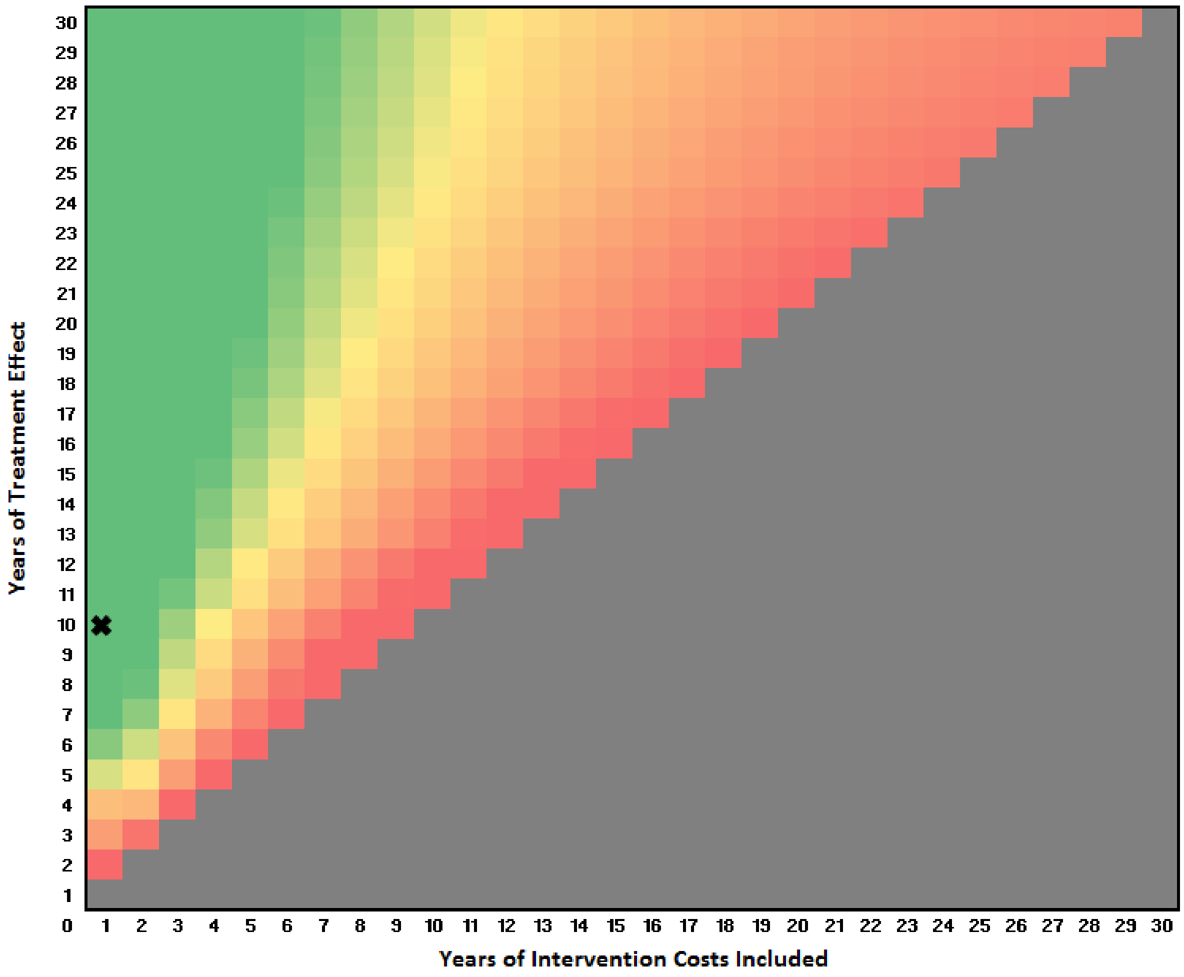

eFigure 1. Two-Way Sensitivity Analysis Showing the ICER for the Shared Incentives Strategy Compared to the Trial Control for Different Combinations of LDL Reduction Waning and Years of Intervention Costs, Assuming a Cost-effectiveness Threshold of $150,000/QALY. The green regions show combinations of values that resulted in an ICER <$100,000/QALY for the shared incentives strategy compared to the trial control strategy; yellow indicates an ICER of $150,000/QALY, red indicates an ICER of >$200,000/QALY; orange indicates an ICER between $150,000-200,000/QALY; gray indicates implausible results (years where intervention costs are included but treatment effects are not observed in those years). “X” marks the base-case assumption and result (treatment effect linearly wanes to zero by year 10).

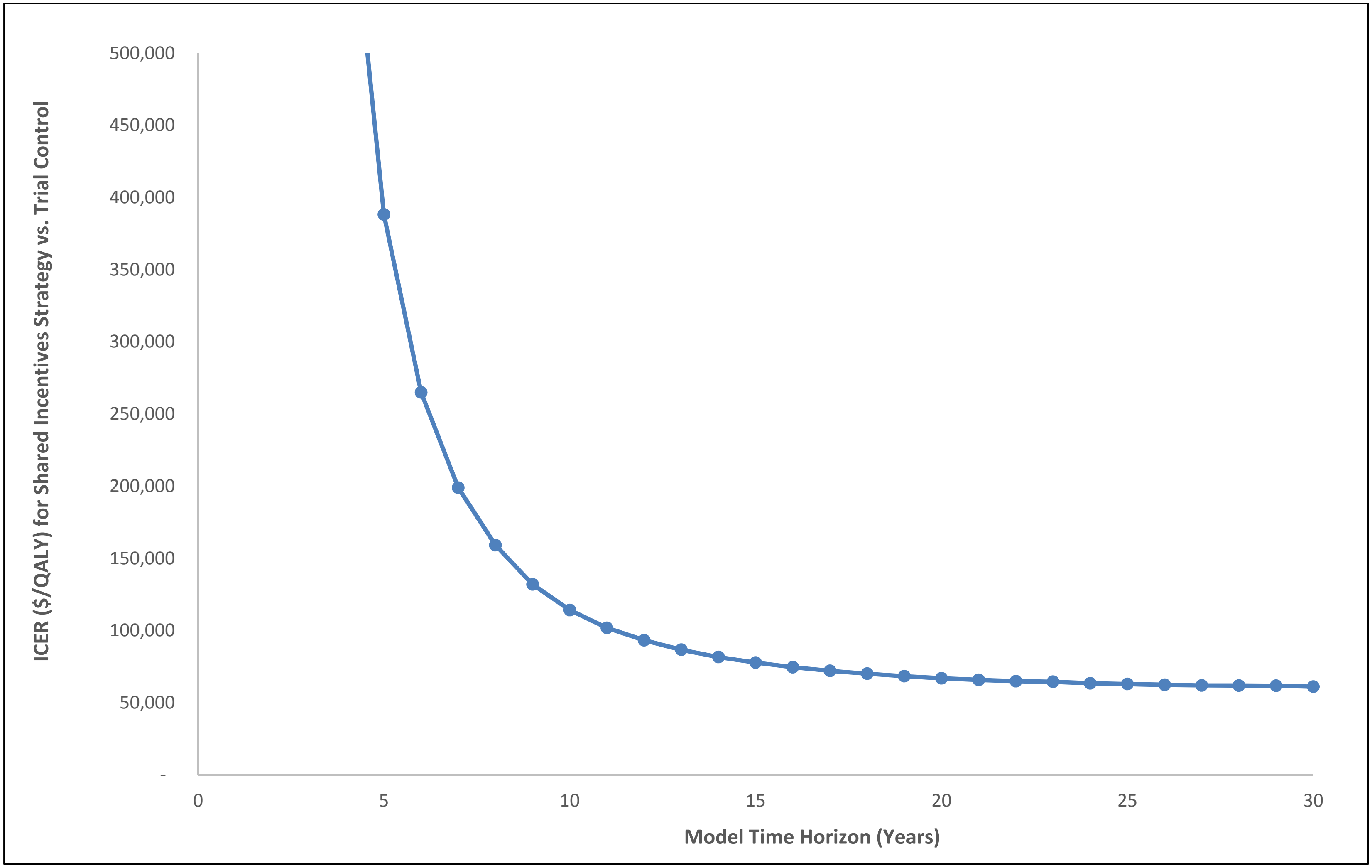

eFigure 2. One-Way Sensitivity Analysis Showing the ICER for the Shared Incentives Strategy as a Function of Analytical Time Horizon.

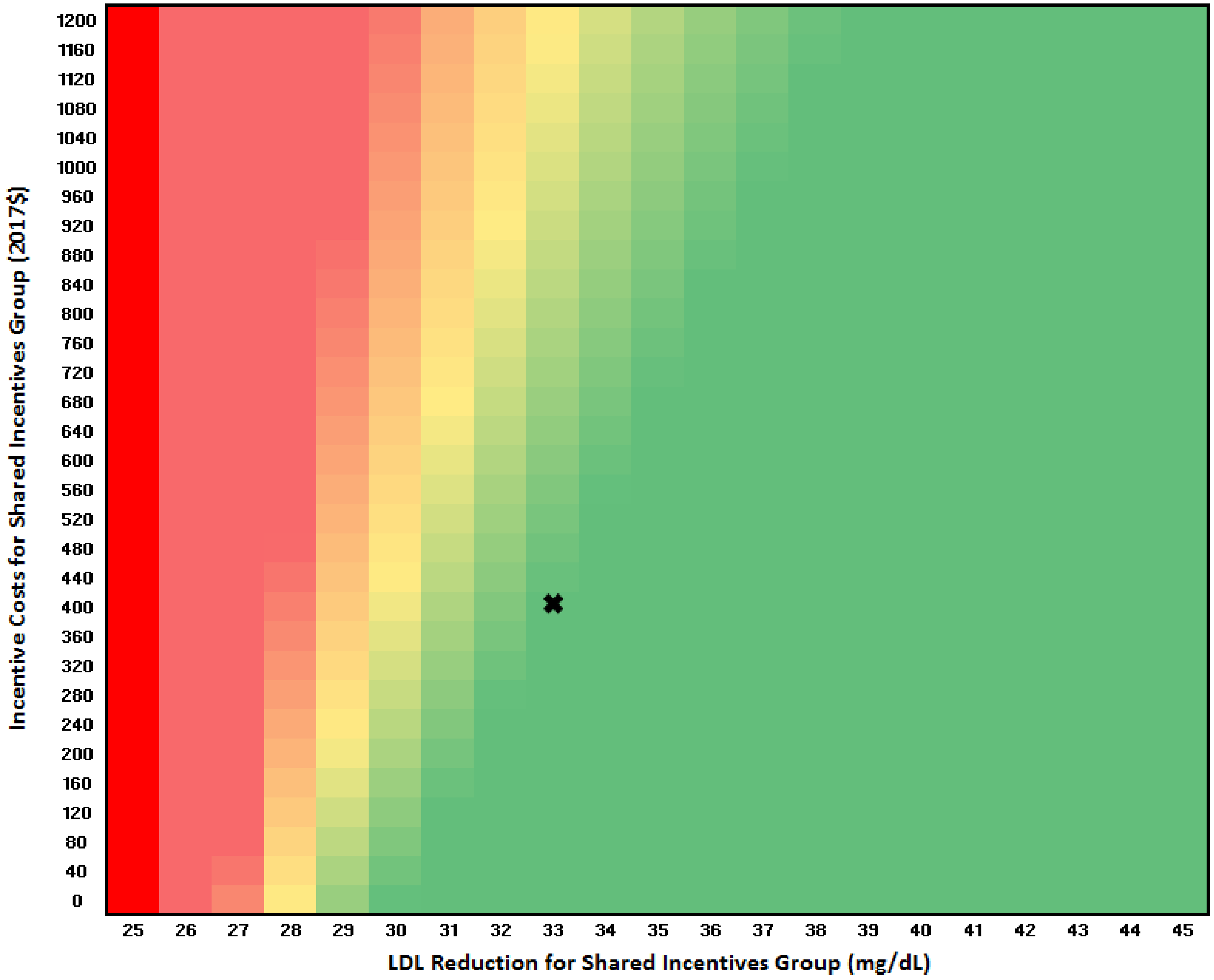

eFigure 3. Two-Way Sensitivity Analysis Showing the ICER for the Shared Incentives Strategy Compared to the Trial Control for Different Combinations of LDL Cholesterol Reductions and Average Shared Financial Incentives Payouts. The green regions show combinations of values that resulted in an ICER <$50,000/QALY for the shared incentives strategy compared to the trial control strategy; yellow indicates an ICER of $100,000/QALY, red indicates an ICER of >$200,000/QALY; orange indicates an ICER between $100,000-200,000/QALY;. "X" marks the base-case assumption and result.

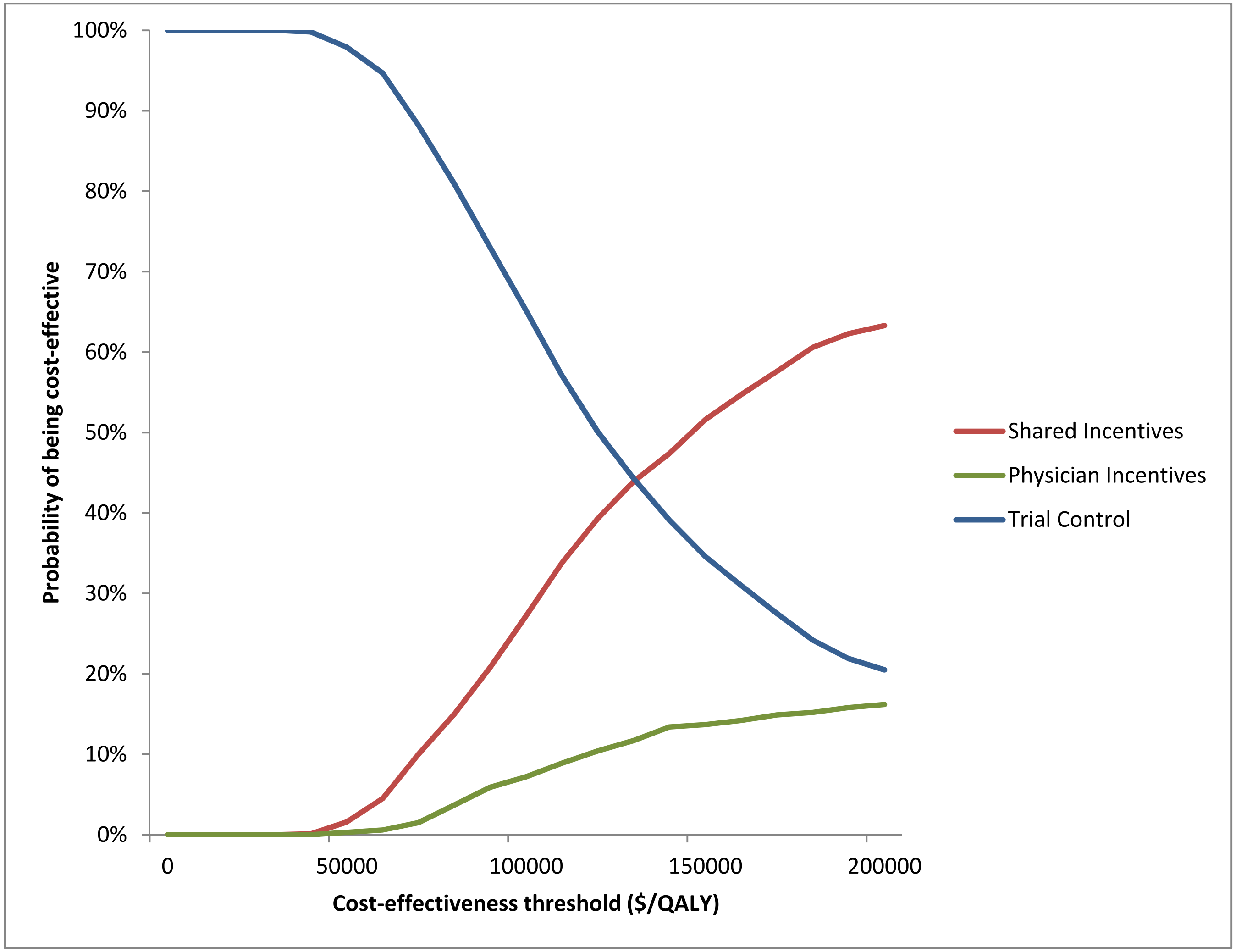

eFigure 4. Cost-effectiveness Acceptability Curve (CEAC) for the Probabilistic Sensitivity Analysis (PSA) for Scenario of 5-Year LDL Reduction Waning.

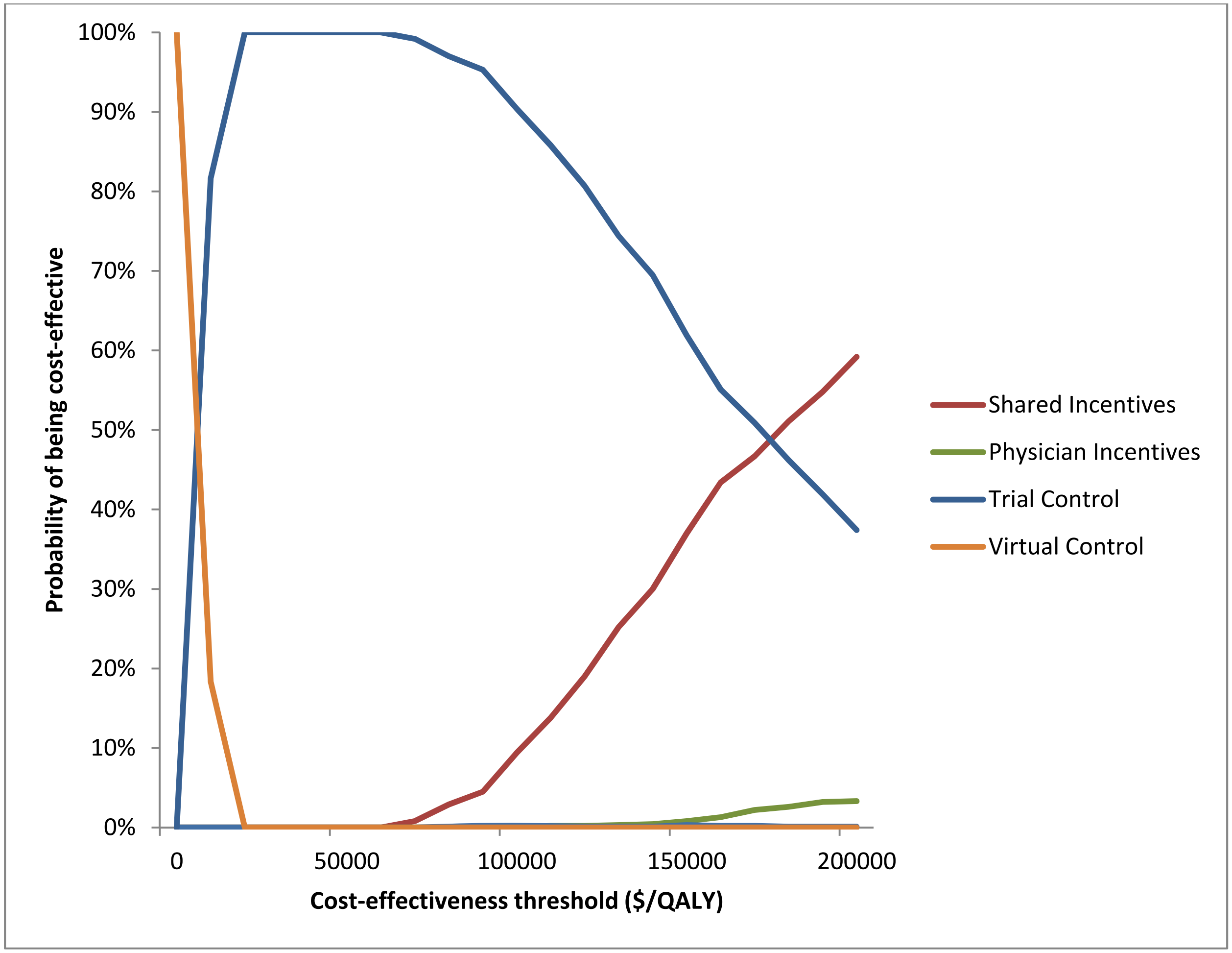

eFigure 5. Cost-effectiveness Acceptability Curve (CEAC) for the Probabilistic Sensitivity Analysis (PSA) for Scenario of 10-Year LDL Reduction Waning and Including 5 Years of Intervention Costs.

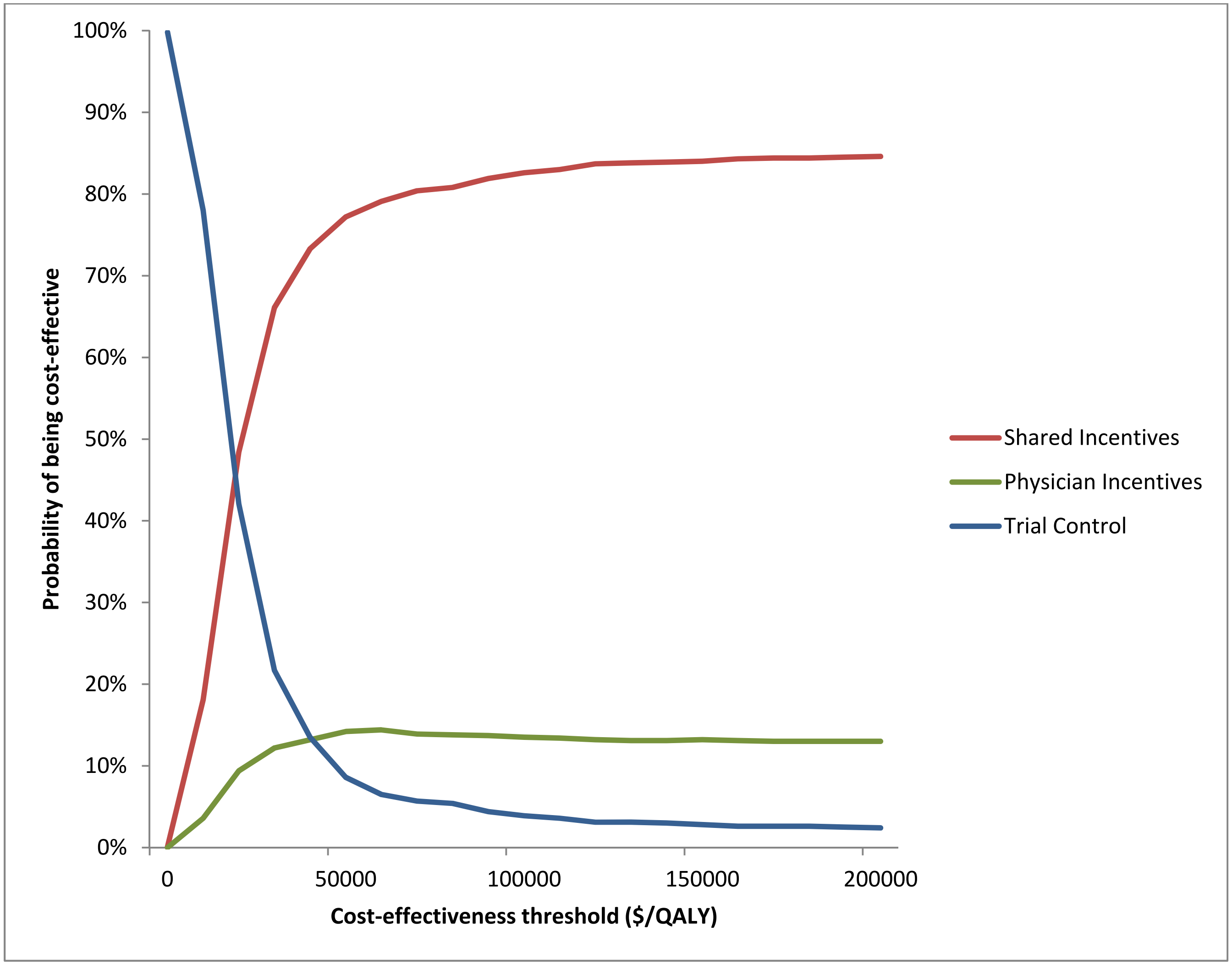

eFigure 6. Cost-effectiveness Acceptability Curve (CEAC) for the Probabilistic Sensitivity Analysis (PSA) for Scenario of 30-Year LDL Reduction Waning

.

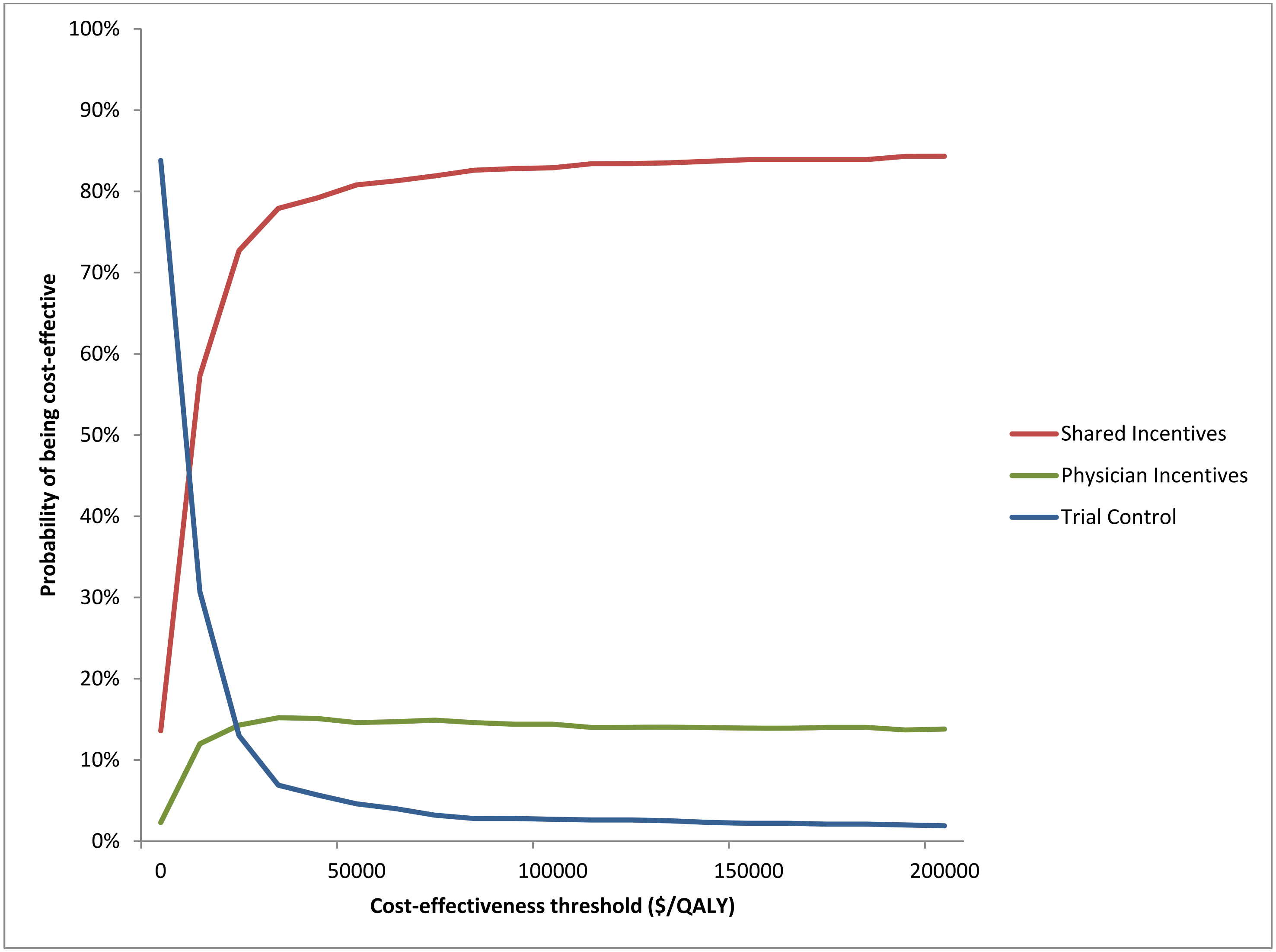

eFigure 7. Cost-effectiveness Acceptability Curve (CEAC) for the Probabilistic Sensitivity Analysis (PSA) for Scenario of Lifetime LDL Benefit Duration.

eReferences

1. Heron M. *Deaths: Leading causes for 2006.* Hyattsville, MD: National Center for Health Statistics;2010.
2. Wolf PA, D'Agostino RB, Belanger AJ, Kannel WB. Probability of stroke: a risk profile from the Framingham Study. *Stroke.* Mar 1991;22(3):312-318.
3. Anderson KM, Odell PM, Wilson PW, Kannel WB. Cardiovascular disease risk profiles. *Am Heart J.* Jan 1991;121(1 Pt 2):293-298.
4. Weinstein MC, Coxson PG, Williams LW, Pass TM, Stason WB, Goldman L. Forecasting coronary heart disease incidence, mortality, and cost: the Coronary Heart Disease Policy Model. *American journal of public health.* Nov 1987;77(11):1417-1426.
5. National Heart Lung and Blood Institute. *Incidence and Prevalence: Chart Book on Cardiovascular and Lung Diseases*: National Institutes of Health;2006.
6. Carandang R, Seshadri S, Beiser A, et al. Trends in incidence, lifetime risk, severity, and 30-day mortality of stroke over the past 50 years. *Jama.* Dec 27 2006;296(24):2939-2946.
7. Touze E, Varenne O, Chatellier G, Peyrard S, Rothwell PM, Mas JL. Risk of myocardial infarction and vascular death after transient ischemic attack and ischemic stroke: a systematic review and meta-analysis. *Stroke; a journal of cerebral circulation.* Dec 2005;36(12):2748-2755.
8. Mozaffarian D, Benjamin EJ, Go AS, et al. Heart Disease and Stroke Statistics-2016 Update: A Report From the American Heart Association. *Circulation.* Jan 26 2016;133(4):e38-e360.
9. Fang J, Alderman MH, Keenan NL, Ayala C. Acute myocardial infarction hospitalization in the United States, 1979 to 2005. *Am J Med.* Mar 2010;123(3):259-266.
10. Williams DO, Abbott JD, Kip KE. Outcomes of 6906 Patients Undergoing Percutaneous Coronary Intervention in the Era of Drug-Eluting Stents. *Report of the DEScover Registry.* 2006;114(20):2154-2162.
11. Capewell S, Murphy NF, MacIntyre K, et al. Short-term and long-term outcomes in 133,429 emergency patients admitted with angina or myocardial infarction in Scotland, 1990-2000: population-based cohort study. *Heart.* Nov 2006;92(11):1563-1570.
12. Windecker S, Stortecky S, Stefanini GG, et al. Revascularisation versus medical treatment in patients with stable coronary artery disease: network meta-analysis. *BMJ : British Medical Journal.* 2014;348.
13. Jokhadar M, Jacobsen SJ, Reeder GS, Weston SA, Roger VL. Sudden death and recurrent ischemic events after myocardial infarction in the community. *American journal of epidemiology.* Jun 1 2004;159(11):1040-1046.
14. Peterson ED, Coombs LP, DeLong ER, Haan CK, Ferguson TB. Procedural volume as a marker of quality for CABG surgery. *JAMA : the journal of the American Medical Association.* Jan 14 2004;291(2):195-201.
15. Yusuf S, Zucker D, Peduzzi P, et al. Effect of coronary artery bypass graft surgery on survival: overview of 10-year results from randomised trials by the Coronary Artery Bypass Graft Surgery Trialists Collaboration. *Lancet.* Aug 27 1994;344(8922):563-570.
16. Hsia J, Jablonski KA, Rice MM, et al. Sudden cardiac death in patients with stable coronary artery disease and preserved left ventricular systolic function. *Am J Cardiol.* Feb 15 2008;101(4):457-461.
17. Hemingway H, Shipley M, Britton A, Page M, Macfarlane P, Marmot M. Prognosis of angina with and without a diagnosis: 11 year follow up in the Whitehall II prospective cohort study. *BMJ (Clinical research ed.).* Oct 18 2003;327(7420):895.
18. Ford ES, Ajani UA, Croft JB, et al. Explaining the Decrease in U.S. Deaths from Coronary Disease, 1980-2000. *New England Journal of Medicine.* 2007;356(23):2388-2398.

19. Nichol G, Thomas E, Callaway CW, et al. Regional variation in out-of-hospital cardiac arrest incidence and outcome. *JAMA : the journal of the American Medical Association.* Sep 24 2008;300(12):1423-1431.

20. Rosen VM, Taylor DC, Parekh H, et al. Cost effectiveness of intensive lipid-lowering treatment for patients with congestive heart failure and coronary heart disease in the US. *Pharmacoeconomics.* 2010;28(1):47-60.

21. Smolina K, Wright FL, Rayner M, Goldacre MJ. Long-term survival and recurrence after acute myocardial infarction in England, 2004 to 2010. *Circ Cardiovasc Qual Outcomes.* Jul 1 2012;5(4):532-540.

22. Sullivan PW, Ghushchyan V. Preference-Based EQ-5D index scores for chronic conditions in the United States. *Med Decis Making.* Jul-Aug 2006;26(4):410-420.

23. Taylor DC, Pandya A, Thompson D, et al. Cost-effectiveness of intensive atorvastatin therapy in secondary cardiovascular prevention in the United Kingdom, Spain, and Germany, based on the Treating to New Targets study. *Eur J Health Econ.* Jul 2009;10(3):255-265.

24. Gage BF, Cardinalli AB, Owens DK. The effect of stroke and stroke prophylaxis with aspirin or warfarin on quality of life. *Arch Intern Med.* Sep 9 1996;156(16):1829-1836.

25. Hutchins R, Viera AJ, Sheridan SL, Pignone MP. Quantifying the utility of taking pills for cardiovascular prevention. *Circ Cardiovasc Qual Outcomes.* Mar 2015;8(2):155-163.

26. Lee KK, Cipriano LE, Owens DK, Go AS, Hlatky MA. Cost-effectiveness of using high-sensitivity C-reactive protein to identify intermediate- and low-cardiovascular-risk individuals for statin therapy. *Circulation.* Oct 12 2010;122(15):1478-1487.

27. Pignone M, Earnshaw S, Tice JA, Pletcher MJ. Aspirin, statins, or both drugs for the primary prevention of coronary heart disease events in men: a cost-utility analysis. *Ann Intern Med.* Mar 7 2006;144(5):326-336.

28. O'Sullivan AK, Rubin J, Nyambose J, Kuznik A, Cohen DJ, Thompson D. Cost estimation of cardiovascular disease events in the US. *Pharmacoeconomics.* Aug 2011;29(8):693-704.

29. Pletcher MJ, Lazar L, Bibbins-Domingo K, et al. Comparing impact and cost-effectiveness of primary prevention strategies for lipid-lowering. *Ann Intern Med.* Feb 17 2009;150(4):243-254.

30. Lazar LD, Pletcher MJ, Coxson PG, Bibbins-Domingo K, Goldman L. Cost-effectiveness of statin therapy for primary prevention in a low-cost statin era. *Circulation.* Jul 12 2011;124(2):146-153.

31. *Red Book*. Montvale (NJ): Thomson Helathcare Inc.; 2009.

32. Nuckols TK, Aledort JE, Adams J, et al. Cost implications of improving blood pressure management among U.S. adults. *Health services research.* Aug 2011;46(4):1124-1157.

33. Shah ND, Mason J, Kurt M, et al. Comparative Effectiveness of Guidelines for the Management of Hyperlipidemia and Hypertension for Type 2 Diabetes Patients. *PLoS ONE.*6(1):e16170.
